# Results from the IMpower132 China cohort: Atezolizumab plus platinum‐based chemotherapy in advanced non‐small cell lung cancer

**DOI:** 10.1002/cam4.5144

**Published:** 2022-09-02

**Authors:** Shun Lu, Jian Fang, Ziping Wang, Yun Fan, Yunpeng Liu, Jianxing He, Jianying Zhou, Jie Hu, Jinjing Xia, Wenxin Liu, Jane Shi, Jing Yi, Lejie Cao

**Affiliations:** ^1^ Shanghai Chest Hospital Shanghai Jiao Tong University Shanghai China; ^2^ Beijing Cancer Hospital Beijing China; ^3^ Key Laboratory of Carcinogenesis and Translational Research (Ministry of Education/Beijing), Department of Thoracic Medical Oncology Peking University Cancer Hospital & Institute Beijing China; ^4^ Zhejiang Cancer Hospital Hangzhou China; ^5^ First Hospital, China Medical University Shenyang China; ^6^ Guangzhou Medical University, First Hospital Guangzhou China; ^7^ Zhejiang University School of Medicine, First Hospital Hangzhou China; ^8^ Zhongshan Hospital, Fudan University Shanghai China; ^9^ F. Hoffmann‐La Roche Ltd Shanghai China; ^10^ Genentech Inc South San Francisco California USA; ^11^ Anhui Provincial Hospital Hefei China; ^12^ Erasca, Inc. South San Francisco California USA

**Keywords:** chemotherapy, China, non‐small cell lung cancer, PD‐L1 inhibitor, PD‐L1 protein

## Abstract

**Background:**

The global Phase III IMpower132 study evaluating atezolizumab plus pemetrexed and carboplatin or cisplatin (APP) versus pemetrexed plus carboplatin or cisplatin (PP) for first‐line treatment of non‐squamous advanced non‐small cell lung cancer (NSCLC) met its co‐primary progression‐free survival (PFS) endpoint at the primary analysis in the intention‐to‐treat (ITT) population. Although the co‐primary overall survival (OS) endpoint was not met, numerical OS improvement favoring APP over PP was observed at the final analysis. We report primary results for Chinese patients in IMpower132.

**Methods:**

Treatment‐naive Chinese patients with non‐squamous stage IV *EGFR*/*ALK* mutation‐negative NSCLC were randomized 1:1 to receive 4 or 6 cycles of APP or PP, followed by maintenance atezolizumab plus pemetrexed or pemetrexed. Co‐primary endpoints were investigator‐assessed PFS and OS.

**Results:**

The ITT population included 163 Chinese patients (82 in the APP arm and 81 in the PP arm). At data cutoff (median follow‐up, 11.7 months), the median PFS in the APP and PP arms was 8.3 and 5.8 months, respectively; the unstratified hazard ratio (HR) was 0.73 (95% CI: 0.50, 1.08). At the interim OS analysis, median OS was not estimable in either arm; the unstratified HR was 0.70 (95% CI: 0.40, 1.24). No new safety signals were observed.

**Conclusion:**

Among Chinese patients in IMpower132, PFS benefit was seen with APP versus PP. Though interim OS data were immature, there was a trend toward OS benefit favoring APP versus PP. The safety profile of the APP was consistent with the known risks of the individual treatment components. ClinicalTrials.gov: NCT02657434.

## Graphical abstract

Atezolizumab plus pemetrexed and cisplatin (APP) versus pemetrexed plus cisplatin (PP) was studied in Chinese patients with non‐squamous advanced non‐small cell lung cancer in IMpower132. Progression‐free survival benefit was seen with APP versus PP; there was a trend toward overall survival benefit favoring APP versus PP, though interim overall survival data were immature at this analysis. No new safety signals were seen with APP in Chinese patients.

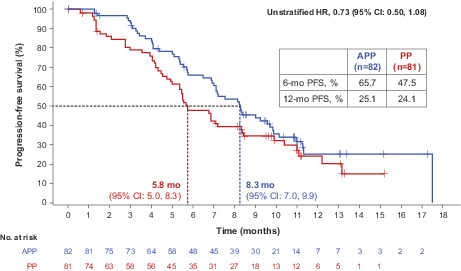

## 1 INTRODUCTION

Despite recent advances in treatment options, lung cancer remains the leading cause of cancer deaths worldwide, including in both the United States and China.^1^ Non‐small cell lung cancer (NSCLC) is the predominant subtype of lung cancer and accounts for approximately 80% to 85% of all cases.^2^, ^3^ First‐line standard‐of‐care treatment for patients with NSCLC whose tumors do not harbor driver mutations currently includes immune checkpoint inhibitor therapy alone, checkpoint inhibitors in combination with chemotherapy—with or without bevacizumab, and dual checkpoint inhibitor combination therapy.^4^, ^5^, ^6^, ^7^, ^8^, ^9^, ^10^, ^11^, ^12^ The programmed death‐ligand 1 (PD‐L1) inhibitor atezolizumab has demonstrated efficacy and tolerability alone and in combination with chemotherapy in NSCLC trials and is currently approved as first‐line and second‐line or later treatment for metastatic NSCLC.^6^, ^9^, ^13^, ^14^, ^15^, ^16^

The Phase III IMpower132 study (NCT02657434) is evaluating the efficacy and safety of atezolizumab in combination with pemetrexed and carboplatin or cisplatin in patients with stage IV non‐squamous NSCLC without epidermal growth factor receptor (*EGFR*) or anaplastic lymphoma kinase (*ALK*) gene mutations. The study met its co‐primary endpoint of progression‐free survival (PFS) in the intention‐to‐treat (ITT) population at the primary analysis (HR, 0.60; 95% CI: 0.49, 0.72; *p* < 0.0001).^17^ Although the co‐primary endpoint of overall survival (OS) was not met at the final analysis in the ITT population, numerical improvement in median OS was observed in the experimental versus control arm (17.5 vs. 13.6 months; HR, 0.86; 95% CI: 0.71, 1.06; *p* = 0.15).^18^

In patients with NSCLC treated with systemic therapy, previous studies have reported differences in therapeutic efficacy and adverse events (AEs) between Asian and non‐Asian populations.^19^, ^20^, ^21^, ^22^, ^23^ Therefore, there is interest in investigating demographic‐specific clinical outcomes and toxicity profiles of systemic NSCLC treatment, including immunotherapy, to further guide the development of treatment strategies. An exploratory subpopulation analysis of Japanese patients in IMpower132 showed clinically meaningful improvement in PFS and OS in the experimental versus control arm (PFS: HR, 0.35; 95% CI: 0.21, 0.58; OS: HR, 0.63; 95% CI: 0.36, 1.14), despite differences in the incidence of Grade 3/4 treatment‐related adverse events (TRAEs).^24^

Here, we report primary efficacy and safety data from an independent analysis in Chinese patients in IMpower132 evaluating atezolizumab plus pemetrexed and cisplatin for first‐line treatment of non‐squamous *EGFR*‐ or *ALK*‐negative NSCLC.

## 2 PATIENTS AND METHODS

### 2.1 Trial design and patients

IMpower132 is a global randomized, open‐label, Phase III study (NCT02657434). The study was conducted in line with Good Clinical Practice guidelines and the Declaration of Helsinki, and the study protocol was approved by an independent ethics committee at each study site. All patients provided written informed consent.

Detailed study eligibility and methods have been previously described.^17^ Briefly, patients with histologically or cytologically confirmed stage IV non‐squamous NSCLC with measurable disease per Response Evaluation Criteria in Solid Tumors (RECIST) version 1.1 who had an Eastern Cooperative Oncology Group performance status (ECOG PS) of 0 or 1 and had not received prior treatment for metastatic disease were enrolled in the study. Patients excluded from the study had untreated CNS metastases, tumors harboring sensitizing *EGFR* mutations or *ALK* alterations, autoimmune diseases, or prior treatment with immunotherapy. Provision of tissue samples was not required at enrollment.

A China extension phase of the study was planned with an objective to assess the treatment effects of atezolizumab in Chinese patients and to evaluate the consistency in treatment effects between Chinese patients and the global population. Based on Chinese health authority requirements, patients were to be randomized if at least one patient was enrolled in China during the global enrollment phase. IMpower132 enrolled a total of 578 patients in the global phase including 1 Chinese patient.^17^ Thus, the China extension phase was initiated. Current residency in mainland China, Hong Kong, or Taiwan and Chinese ancestry were required. Chinese patients were enrolled across 21 clinical sites (20 sites in mainland China and 1 site in Taiwan). Since tissue samples were not required at enrollment, tumor PD‐L1 expression data were not collected for Chinese patients.

### 2.2 Treatment

Patients were randomized 1:1 to receive either atezolizumab plus pemetrexed and carboplatin or cisplatin (APP arm) or pemetrexed plus carboplatin or cisplatin (PP arm). Stratification factors for randomization in the global study were sex (male or female), ECOG PS (0 or 1), chemotherapy type (carboplatin vs. cisplatin) and smoking status (never vs. current and/or former). As pemetrexed was only approved in combination with cisplatin for the first‐line treatment of NSCLC at that time, patients in the China extension phase of the study did not receive carboplatin. Therefore, chemotherapy was not a stratification factor for randomization in these patients. Induction treatment was administered in four or six 21‐day cycles, with the number of cycles determined by the investigator prior to randomization. Study treatments were given intravenously on day 1 of each 21‐day cycle at the following doses: atezolizumab 1200 mg, cisplatin 75 mg/m^2^, pemetrexed 500 mg/m^2^. Maintenance therapy was either atezolizumab plus pemetrexed for patients in the APP arm or pemetrexed alone for patients in the PP arm, given every 21 days until unacceptable toxicity, disease progression per RECIST 1.1, or death. Atezolizumab continuation by patients in the APP arm was allowed after disease progression if evidence of clinical benefit as assessed by the investigator was established. Crossover between treatment groups was not allowed.

### 2.3 Outcomes and assessments

The co‐primary endpoints for the study were investigator‐assessed PFS measured per RECIST 1.1 and OS in the ITT population. Key secondary endpoints included investigator‐assessed objective response rate (ORR), duration of response (DOR) per RECIST 1.1, and OS rates at 12 and 24 months.

Tumors were assessed at baseline and then every 6 weeks for the first 48 weeks following cycle 1 of day 1. This was followed by assessments every 9 weeks until radiographic disease progression per RECIST 1.1 or loss of clinical benefit (for patients in the APP arm who continued atezolizumab treatment after radiologic disease progression per RECIST 1.1), withdrawal of consent, death, or study termination by sponsor, whichever occurred first.

The safety and tolerability of the study medicines were evaluated by monitoring the incidence, severity, and nature of AEs according to the National Cancer Institute Common Terminology Criteria for Adverse Events (version 4.0) in the safety‐evaluable population, defined as patients who received any amount of the study treatment.

### 2.4 Statistical analysis

Detailed description of the statistical analyses for the global population of IMpower132 have been previously reported.^17^ In brief, the study was designed to enroll approximately 568 patients in the global enrollment phase based on the number of events required to demonstrate statistical difference in both PFS and OS between the APP and PP arms. It was planned that the primary PFS analysis would be conducted after approximately 458 recorded PFS events in the ITT population and at least 10 months after enrollment of the last patient, whichever occurred last. An interim OS analysis was planned to be conducted along with the primary PFS analysis for the global ITT population. The final OS analysis was planned to be conducted after 398 OS events had occurred in the global ITT population. Comparisons between the APP versus PP arms regarding PFS and OS were tested based on a stratified log‐rank test, with stratification factors being sex (male vs. female), ECOG PS (0 vs. 1), and chemotherapy regimen (carboplatin vs. cisplatin). A stratified Cox regression model was used to estimate the HRs and 95% CIs. Median PFS and OS for the APP and PP arms were estimated using Kaplan–Meier methodology, and survival curves were constructed for visual descriptions of the difference between the two treatment arms. For the construction of the 95% CI for the median PFS and OS, the Brookmeyer‐Crowley method was employed. ORR and its 95% CI were calculated using the Clopper‐Pearson method, while DOR was estimated using Kaplan–Meier methodology.

Analyses involving Chinese patients were done separately from those involving patients enrolled during the global enrollment phase; thus, Chinese patients in the study represented a patient population independent of the global study. The final PFS and OS analyses in Chinese patients were planned to be conducted after approximately 115 recorded PFS events and approximately 120 recorded OS events had occurred in the ITT population. The planned 115 PFS events would provide 89% probability of observing at least 50% of the risk reduction in PFS expected to be observed in the global population. Determination of median PFS, OS, DOR, ORR, and construction of 95% CIs was performed as described for the global population, although unstratified analyses were performed in Chinese patients, as pre‐defined in the statistical analysis plan, to avoid over‐stratification in this smaller sample size. The analyses in Chinese patients were not powered to determine statistical significance.

Safety was evaluated in all Chinese patients who received any amount of any of the study medicines; safety results are presented descriptively. AE of special interest (AESI) terms were specified by the study sponsor, regardless of investigator‐assessed causality. Statistical analyses were conducted with SAS version 9.4.

## 3 RESULTS

### 3.1 Participants

**FIGURE 1:**
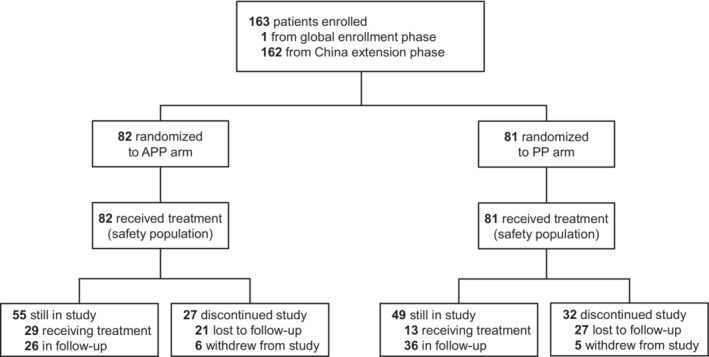
Disposition of Chinese patients in IMpower132. One patient from Taiwan from the global enrollment phase was included. APP, atezolizumab plus cisplatin plus pemetrexed; PP, cisplatin plus pemetrexed.

A total of 163 Chinese patients (ITT population) were enrolled between May 31, 2017, and November 9, 2018. The ITT population comprised 1 patient from the global enrollment phase who originated from Taiwan and 162 patients from mainland China in the China extension phase. Following randomization, 82 patients received APP and 81 patients received PP. At the data cutoff (July 18, 2019), the median follow‐up was 11.7 months, with a minimum follow‐up of 8.3 months; 104 patients (63.8%) were still in the study, including 55 patients (67.1%) in the APP arm and 49 patients (60.5%) in the PP arm. Fifty‐nine patients (36.2%) discontinued from the study: 27 (32.9%) in the APP arm and 32 (39.5%) in the PP arm (Figure 1). Baseline characteristics were generally balanced between treatment arms, although a slightly higher proportion of patients in the APP arm (*n* = 12, 14.6%) had baseline liver metastases than in the PP arm (*n* = 5, 6.2%; Table 1 **)**.

**TABLE 1:** Baseline demographic and clinical characteristics

	APP (*n* = 82)	PP (*n* = 81)
Age, median (range), year	61.0 (32–77)	61.0 (33–80)
Age group		
<65 years	57 (69.5)	55 (67.9)
≥65 years	25 (30.5)	26 (32.1)
Sex		
Male	60 (73.2)	59 (72.8)
Female	22 (26.8)	22 (27.2)
Race		
Asian	82 (100)	81 (100)
Tobacco use history		
Never	30 (36.6)	27 (33.3)
Current or former	52 (63.4)	54 (66.7)
ECOG PS		
0	22 (26.8)	22 (27.2)
1	60 (73.2)	59 (72.8)
Liver metastasis		
Yes	12 (14.6)	5 (6.2)
No	70 (85.4)	76 (93.8)
Intended no. of chemotherapy cycles at induction		
4	53 (64.6)	50 (61.7)
6	29 (35.4)	31 (38.3)
Non‐squamous histology detail		
n	82	80
Adenocarcinoma	76 (92.7)	78 (97.5)
Adenocarcinoma with neuroendocrine features	3 (3.7)	0
Adenosquamous	0	2 (2.5)
Not applicable	3 (3.7)	0
*EML4‐ALK* rearrangement status		
Negative	82 (100)	81 (100)
*EGFR* mutation status		
Positive^a^	1 (1.2)	5 (6.2)
Negative	81 (98.8)	76 (93.8)
*KRAS* mutation status		
Positive	2 (2.4)	3 (3.7)
Negative	34 (41.5)	32 (39.5)
Unknown	46 (56.1)	46 (56.8)

*Note*: Data are n (%) except where otherwise indicated. Data cutoff was July 18, 2019.

Abbreviations: APP, atezolizumab plus cisplatin plus pemetrexed; ECOG PS, Eastern Cooperative Oncology Group performance status; PP, cisplatin plus pemetrexed.

^a^ All 6 patients had tumors harboring non‐sensitizing *EGFR* exon 20 insertion mutations.

### 3.2 Efficacy

**FIGURE 2:**
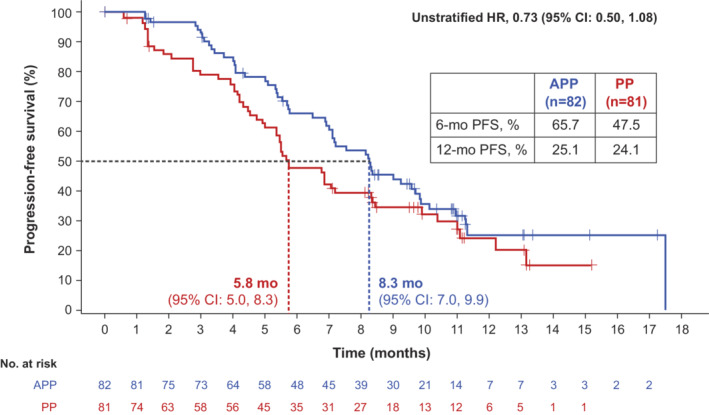
Investigator‐assessed progression‐free survival (PFS) at the primary PFS analysis. Data cutoff was July 18, 2019. APP, atezolizumab plus cisplatin plus pemetrexed; PP, cisplatin plus pemetrexed.

At the primary PFS analysis, a total of 52 PFS events (63.4%) had occurred in the APP arm and 54 events (66.7%) had occurred in the PP arm. Median PFS was 8.3 months with APP versus 5.8 months with PP (unstratified HR, 0.73; 95% CI: 0.50, 1.08). In the APP and PP arms, landmark PFS rates were 65.7% and 47.5% at 6 months and 25.1% and 24.1% at 12 months, respectively (Figure 2). PFS for patient subgroups is shown in Figure S1.

**FIGURE 3:**
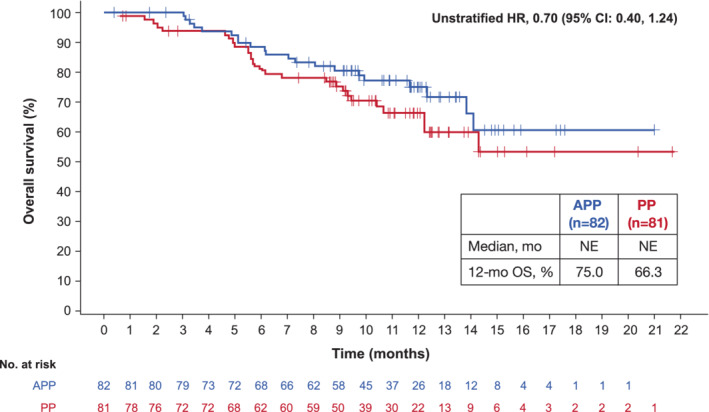
Overall survival (OS) at the interim OS analysis. Data cutoff was July 18, 2019. APP, atezolizumab plus cisplatin plus pemetrexed; NE, not evaluable; PP, cisplatin plus pemetrexed.

At the interim OS analysis, which was conducted at the time of the primary PFS analysis, a total of 21 OS events (25.6%) had occurred in the APP arm and 27 OS events (33.3%) had occurred in the PP arm. Median OS was not estimable in either arm at the interim OS analysis (unstratified HR, 0.70; 95% CI: 0.40, 1.24; Figure 3). Landmark OS rates at 12 months were 75.0% in the APP arm and 66.3% in the PP arm (Figure 3).

Overall, 30.5% of patients (*n* = 25) in the APP arm and 39.5% of patients (*n* = 32) in the PP arm received ≥1 subsequent non‐protocol therapy. No patients in the APP arm and 10 patients in the PP arm (12.3%) received ≥1 subsequent non‐protocol immunotherapy, with the most common agents being nivolumab (*n* = 5, 6.2%) and pembrolizumab (*n* = 3; 3.7%; Table S1).

At the data cutoff, the confirmed ORR was 56.1% (95% CI: 44.7, 67.0) in the APP arm and 27.2% (95% CI: 17.9, 38.2) in the PP arm, with a difference of 28.9% (95% CI: 13.3, 44.6). The median duration of response was 6.7 months (95% CI: 5.7, 8.0) in the APP arm and 8.5 months (95% CI: 4.2, not evaluable) in the PP arm (Table 2).

**TABLE 2:** Confirmed objective response rate and duration of response

	APP (*n* = 82)	PP (*n* = 81)
Objective response rate	46 (56.1)	22 (27.2)
Difference (95% CI), %	28.9 (13.3, 44.6)	
Complete response	0	0
Partial response	46 (56.1)	22 (27.2)
Stable disease	27 (32.9)	40 (49.4)
Progressive disease	4 (4.9)	9 (11.1)
Duration of response, median (95% CI), mo	6.7 (5.7, 8.0)	8.5 (4.2, NE)
Responders without event	16 (34.8)	10 (45.5)

*Note*: Data are n (%) except where otherwise indicated. Five patients in the APP arm and 10 patients in the PP arm were classified as missing or unevaluable for response. Data cutoff was July 18, 2019.

Abbreviations: APP, atezolizumab plus cisplatin plus pemetrexed; NE, not evaluable; PP, cisplatin plus pemetrexed.

### 3.3 Safety

The safety‐evaluable population included 82 patients in the APP arm and 81 patients in the PP arm. At the data cutoff, the median duration of treatment in the APP arm was 6.7 months with atezolizumab (range, 0–17 months), 5.9 months with pemetrexed (range, 0–17 months), and 2.2 months (range, 0–4 months) with cisplatin. The median treatment duration in the PP arm was 4.7 months (range, 0–15 months) with pemetrexed and 2.1 months (range, 0–4 months) with cisplatin (Table S2).

All‐grade AEs of any cause occurred in 81 (98.8%) and 78 (96.3%) of patients in the APP and PP arms, respectively (Table 3). All‐cause AEs with an incidence rate of ≥10% in any arm are shown in Table S3. TRAEs occurred in 97.6% and 91.4% of patients, respectively, with the most common being decreased neutrophil count (APP, 80.5%; PP, 55.6%), decreased white blood cell count (APP, 72.0%; PP, 56.8%), anemia (APP, 75.6%; PP, 66.7%), and nausea (APP, 48.8%; PP, 50.6%). Grade 3/4 TRAEs were reported in 62.2% of APP‐treated patients and 45.7% of PP‐treated patients (Table 3). Grade 5 TRAEs were pneumonia (*n* = 1; 1.2%) and lung infection (*n* = 1; 1.2%) in the APP arm and lung infection (*n* = 1; 1.2%) and bone marrow failure (*n* = 1; 1.2%) in the PP arm. Serious AEs of any grade were reported in 39.0% and 28.4% of patients in the APP and PP arm, respectively, and were treatment related in 31.7% and 17.3% of patients, respectively. AEs leading to treatment withdrawal occurred in 15.9% of patients in the APP arm and 19.8% of patients in the PP arm. AESIs were reported in 74.4% and 42.0% of patients in the APP and PP arms, respectively, and were grade 3/4 severity in 9.8% and 6.2% of patients, respectively (Table 3). Hepatitis was the most common any‐grade AESI, occurring in 53.7% of the APP arm and 30.9% of the PP arm; most events were laboratory result abnormalities (52.4% and 30.9%, respectively). Grade 3/4 AESIs were hepatitis laboratory abnormalities (APP, 2.4%; PP, 3.7%), rash (APP, 3.7%; PP, 1.2%), pneumonitis (APP, 1.2%; PP, 1.2%), hepatitis diagnosis (APP, 1.2%; PP, 0%), and infusion‐related reaction (APP, 1.2%; PP, 0%; Table S4). Only one grade 5 AESI was reported (PP arm, immune‐mediated hepatitis [laboratory abnormalities]).

**TABLE 3:** Summary of safety among treated patients

Patients, n (%)	APP (*n* = 82)	PP (*n* = 81)
Any‐grade AE	81 (98.8)	78 (96.3)
Treatment related	80 (97.6)	74 (91.4)
Grade 3/4 AE	54 (65.9)	38 (46.9)
Treatment related	51 (62.2)	37 (45.7)
Serious AE	32 (39.0)	23 (28.4)
Treatment related	26 (31.7)	14 (17.3)
Grade 5 AE	5 (6.1)	7 (8.6)
Treatment related	2 (2.4)	2 (2.5)
AE leading to any treatment withdrawal	13 (15.9)	16 (19.8)
AE leading to any dose modification or interruption	35 (42.7)	21 (25.9)
Atezo AESI	61 (74.4)	34 (42.0)
Grade 3/4	8 (9.8)	5 (6.2)

*Note*: Treatment‐related AEs are related to any component of treatment. Data cutoff was July 18, 2019.

Abbreviations: AE, adverse event; AESI, AE of special interest; APP, atezolizumab plus cisplatin plus pemetrexed; atezo, atezolizumab; PP, cisplatin plus pemetrexed.

## 4 DISCUSSION

In this independent analysis of data from Chinese patients in IMpower132, APP demonstrated PFS benefit versus PP. Interim OS data for these patients were not mature at this data cut; however, the OS HR point estimate favored APP over PP, and the OS rate at 12 months was higher in the APP arm than in the PP arm. Confirmed ORR was higher in the APP arm than in the PP arm; however, median DOR was longer with PP than with APP.

A patient population independent of the global study comprised the China cohort, although 1 patient of Chinese ancestry from Taiwan who was enrolled in the global study was included in the analysis. While outcomes were largely consistent, differences in the prevalence (>10%) of some baseline characteristics were seen between the China cohort (Table 1) and the global population.^17^ The China cohort had a lower prevalence of patients age ≥65 years (31% in the China cohort vs. 45% in the global population), a higher prevalence of never smokers (35% vs. 12%), a lower prevalence of current or former smokers (65% vs. 88%), a lower prevalence of patients with an ECOG PS of 0 (27% vs. 42%), and a higher prevalence of patients with an ECOG PS of 1 (73% vs. 58%) than in the global population. While fewer than half of the China cohort was assessed for *KRAS* mutation status, the prevalence among those tested was lower in the China cohort (7%) than the global population (28%).^17^ Finally, tumor PD‐L1 expression level was not assessed in the China cohort, but tissue was available for ≈60% of patients in the global population, of which 53% of patients were tumor PD‐L1 positive.^17^

Similar observations regarding efficacy in the China cohort were seen in the global population. The co‐primary PFS endpoint was met in the global ITT population at the primary analysis (HR, 0.60; 95% CI: 0.49, 0.72; *p* < 0.0001), with consistent PFS benefit also observed with APP compared with PP at the updated PFS analysis (HR, 0.56; 95% CI: 0.47, 0.67; *p* < 0.0001).^18^ Although the significance boundary for the co‐primary OS endpoint was not crossed at the final OS analysis in the global ITT population, OS was longer in the APP arm than in the PP arm (HR, 0.86; 95% CI: 0.71, 1.06).^17^ In both Chinese patients and the global population, more patients in the PP arm than the APP arm were treated with subsequent non‐protocol therapies, including immunotherapy. Among patients in the global population, 6% of the APP arm and 46% of the PP arm received subsequent non‐protocol immunotherapy at the final analysis. While treatment with subsequent non‐protocol immunotherapy may have impacted survival outcomes in the global population, additional survival follow‐up is needed to assess the potential impact of non‐protocol therapy on outcomes in Chinese patients. The difference in ORR between the APP and PP arms was higher in Chinese patients (28.9%) than in the global population (14.7%), although results were generally comparable between Chinese and global patients.^17^ The observation that the DOR was longer in the PP arm than the APP arm is difficult to interpret and may be due to the limited number of ORR events in the China cohort.

Previous studies have reported differences in efficacy and safety between Asian and non‐Asian populations with NSCLC treated with systemic therapy.^19^, ^20^, ^21^, ^22^, ^23^ In a meta‐analysis of pooled data from 11 metastatic NSCLC trials that compared survival outcomes in Asian versus non‐Asian patients, Asian patients appeared to have a better prognosis than non‐Asian patients.^25^ At the primary analysis of IMpower132, subgroup analyses in the global ITT population showed PFS benefit with APP versus PP among Asian patients (HR, 0.42; 95% CI: 0.28, 0.63).^17^ Notably, median PFS in the APP arm was 10.2 months in the Asian subpopulation and 7.6 months in the global population, with median PFS in the PP arm being 5.3 months (Asian subpopulation) and 5.2 months (global population), which may suggest additional atezolizumab treatment benefit among Asian patients. Further, in an exploratory subpopulation analysis of data from Japanese patients in IMpower132 (final PFS analysis), a similar trend was observed for both PFS and OS, further suggesting that Asian patients may derive additional treatment benefits from atezolizumab.^24^ Consistent with these observations in Asian subpopulations, in Chinese patients from IMpower132, median PFS for the APP arm at the primary analysis was slightly longer than that for the global ITT population at the final PFS analysis, despite 3.1 months of additional follow‐up in the global study.^17^ Of note, median PFS in the PP arm was comparable at the primary analyses of data from Chinese patients and the global population (5.8 vs. 5.2 months, respectively), suggesting added PFS benefit with atezolizumab in Chinese patients.

Results from recently reported Phase III studies evaluating first‐line chemo‐immunotherapy combinations in Chinese patients with advanced non‐squamous NSCLC further support the efficacy observations from our study. In the ORIENT‐11 study comparing sintilimab (anti‐PD‐1) plus pemetrexed and platinum‐based chemotherapy, a median PFS of 8.9 months was observed in the experimental arm compared with 5.0 months in the control arm.^26^ Similarly, the RATIONAL 307 trial evaluating the PD‐1 inhibitor tislelizumab in combination with chemotherapy reported a median PFS of 7.6 versus 5.5 months in the experimental and control arm, respectively.^27^ Another study, the CameL trial assessing camrelizumab (anti‐PD‐1) plus chemotherapy versus chemotherapy alone, reported an interim median PFS of 11.3 and 8.3 months in the experimental and control arms, respectively.^28^ Taken together, data from these studies and those from IMpower132 suggest the effectiveness of PD‐L1/PD‐1 inhibitors in combination with chemotherapy in Chinese populations. Of note, in each of the abovementioned trials, PFS was the primary endpoint, with OS included among the secondary endpoints, which may indicate acknowledgment of the confounding effects of subsequent non‐protocol therapies on OS results in first‐line studies.^29^

In Chinese patients, APP demonstrated a tolerable safety profile consistent with the known risks of the individual treatment components, with no new or unexpected safety signals identified. Any‐cause AEs and TRAEs, including those of Grade 3/4 severity, occurred more frequently in the APP arm than in the PP arm; these observations could be attributed to the longer treatment duration in the APP versus PP arm. Grade 5 AEs and AEs leading to any treatment withdrawal were reported in a higher proportion of patients in the PP arm than the APP arm, further demonstrating the favorable safety profile of APP versus PP. A higher proportion of Chinese patients had AESIs compared with the global population; however, the majority of these events were Grade 1/2 and were mainly driven by the high incidence of low‐grade hepatitis laboratory result abnormalities in the Chinese patients. Notably, this increased incidence of hepatitis was seen in both the APP and PP arms compared with the global population, consistent with a higher prevalence of hepatitis in the general Chinese population than in the populations of other countries globally.^30^, ^31^ A lower proportion of Chinese patients had serious AEs, Grade 5 AEs, and AEs leading to treatment withdrawal compared with the global population. Overall, reported AEs were distributed across multiple organs and systems, with no specific trend observed.

Strengths of this study include its design, which allowed for enrollment and analysis of a Chinese patient population distinct from that of the global population. Limitations of this study include the small number of Chinese patients, lack of PD‐L1 testing, and immaturity of the OS data. While the objective of the China extension phase was met, analyses were not powered to determine statistical significance.

In conclusion, atezolizumab in combination with pemetrexed and cisplatin demonstrated PFS benefit over pemetrexed plus cisplatin at the primary analysis in Chinese patients in IMpower132. Although the interim OS data were not mature, a trend toward OS benefit favoring atezolizumab was observed. No new or unexpected safety signals were identified. To our knowledge, this study is the first Phase III study demonstrating PFS benefit of a PD‐L1 inhibitor, atezolizumab, in combination with platinum‐based doublet chemotherapy for first‐line treatment of advanced non‐in a Chinese population.

## AUTHOR CONTRIBUTIONS

Shun Lu: Conceptualization, Methodology, Validation, Formal analysis, Investigation, Resources, Writing—Original Draft, Writing—Review & Editing, Visualization, Supervision. Lejie Cao: Conceptualization, Investigation, Resources, Writing‐ Review & Editing, Supervision, Project Administration. Jian Fang: Conceptualization, Investigation, Resources, Writing—Review & Editing, Supervision, Project Administration. Ziping Wang: Resources, Writing—Review & Editing. Yun Fan: Conceptualization, Methodology, Investigation, Resources, Writing—Review & Editing. Yunpeng Liu: Conceptualization, Investigation, Resources, Writing—Review & Editing, Supervision, Project Administration. Jianxing He: Conceptualization, Investigation, Resources, Writing—Review & Editing, Supervision, Project Administration. Bangwei Cao: Conceptualization, Investigation, Resources, Writing—Review & Editing, Supervision, Project Administration. Jianying Zhou: Conceptualization, Investigation, Resources, Writing—Review & Editing, Supervision, Project Administration. Jie Hu: Validation, Investigation, Resources, Data Curation, Writing—Review & Editing, Visualization. Jing Yi: Methodology, Writing—Review & Editing, Supervision. Wenxin Liu: Formal analysis, Writing—Review & Editing. Jane Shi: Writing‐ Review & Editing. Jinjing Xia: Writing—Review & Editing.

## FUNDING INFORMATION

IMpower132 was sponsored by F. Hoffmann‐La Roche Ltd/Genentech Inc., a member of the Roche Group.

## CONFLICT OF INTERESTS

Dr Lu reports grants or contracts from AstraZeneca Pharmaceuticals LP, Hutchison MediPharma, Bristol Myers Squibb Company, Jiangsu Hengrui Medicine Co Ltd, Beigene, F. Hoffmann‐La Roche Ltd, and Jiangsu Hansoh Pharmaceutical Co. Ltd; consulting fees from AstraZeneca Pharmaceuticals LP, Pfizer Inc, Boehringer Ingelheim, Hutchison MediPharma, Simcere Pharmaceutical, ZaiLab Ltd, GenomiCare Biotechnology, Yuhan Corporation, prIME Oncology, Menarini Group, InventisBio Inc Ltd, and F. Hoffmann‐La Roche Ltd; honoraria from AstraZeneca Pharmaceuticals LP, F. Hoffmann‐La Roche Ltd, Jiangsu Hansoh Pharmaceutical Co Ltd, and Jiangsu Hengrui Medicine Co Ltd; and served on an advisory board for F. Hoffmann‐La Roche Ltd, AstraZeneca Pharmaceuticals LP, Xcovery Holding, and Regeneron Pharmaceuticals. Drs Xia, W. Liu, and Shi report employment by F. Hoffmann‐La Roche Ltd. Dr Yi reports employment by Genentech Inc and stock ownership. Drs Wang, Fan, Y. Liu, He, Zhou, Hu, and L. Cao report no conflicts of interest to disclose.

## ROLE OF THE FUNDING SOURCE

This study is sponsored by F. Hoffmann‐La Roche Ltd/Genentech Inc, a member of the Roche Group. The sponsor provided the study drugs and support for the trial as well as collaborated with authors on study design, data collection, data analyses, and data interpretation. Editorial support, funded by the sponsor, was provided by an independent medical writer under the guidance of the authors.

## Supporting information


Appendix S1

## Data Availability

For eligible studies, qualified researchers may request access to individual patient‐level clinical data through a data request platform. At the time of writing this request platform is Vivli. https://vivli.org/ourmember/roche/. For up to date details on Roche's Global Policy on the Sharing of Clinical Information and how to request access to related clinical study documents, see here: https://go.roche.com/data_sharing. Anonymized records for individual patients across more than one data source external to Roche cannot, and should not, be linked due to a potential increase in risk of patient re‐identification.
