# Supplementary material for: Results from the IMpower132 China cohort: Atezolizumab plus platinum‐based chemotherapy in advanced non‐small cell lung cancer
Source: Cancer Med. 2022 Sep 2;12(3):2666–76. doi: 10.1002/cam4.5144 (PMC9939192; doi:10.1002/cam4.5144)
Supplement: Supplementary file 1 — Appendix S1 [file CAM4-12-2666-s001.docx]

**SUPPLEMENTAL APPENDIX**

**LIST OF INVESTIGATORS**

The following investigators participated in the IMpower132 China Cohort study

***China:*** B. Cao, L. Cao, Y. Chen, Y. Fan, J. Fang, J. He, J. Hu, Y. Liu, S. Lu, H. Pan, Y. Pan,
Y. Shu, C. Wang, X. Wang, Z. Wang, G. Wu, N. Yang, D. Zhong, J. Zhou, L. Zhu

***Taiwan:*** J.-Y. Shih

**Supplemental Table S1.** Subsequent anti-cancer therapies, by arm

| **Patients, n (%)** | **APP**  **(n=82)** | **PP**  **(n=81)** |
| --- | --- | --- |
| Patients with ≥1 treatment | 25 (30.5) | 32 (39.5) |
| Chemotherapy | 23 (28.0) | 26 (32.1) |
| Targeted therapy | 15 (18.3) | 20 (24.7) |
| Immunotherapy | 0 | 10 (12.3) |
| Nivolumab | 0 | 5 (6.2) |
| Pembrolizumab | 0 | 3 (3.7) |
| Sintilimab | 0 | 1 (1.2) |
| Tislelizumab | 0 | 1 (1.2) |

A patient was counted once if they received >1 non-protocol therapy of the same type. A patient was counted more than once if they received >1 type of non-protocol therapy. Data cutoff was July 18, 2019. APP, atezolizumab plus cisplatin plus pemetrexed; PP, cisplatin plus pemetrexed.

**Supplemental Table S2.** Treatment exposure, by arm

|  | **APP  (n=82)** | | | **PP  (n=81)** | |
| --- | --- | --- | --- | --- | --- |
|  | **Atezo** | **Pem** | **Cis** | **Pem** | **Cis** |
| Treatment duration, median, mo | 6.7 | 5.9 | 2.2 | 4.7 | 2.1 |
| Dose intensity, median, % | 97.0 | 97.0 | 98.8 | 98.3 | 98.8 |
| Total cumulative dose, mg |  |  |  |  |  |
| Mean | 13,169.3 | 8456.2 | 492.5 | 6357.9 | 465.0 |
| Median | 12,000.0 | 7520.0 | 480.8 | 5520.0 | 480.0 |

Chinese patients did not receive carboplatin. Data cutoff was July 18, 2019. APP, atezolizumab plus cisplatin plus pemetrexed; atezo, atezolizumab; cis, cisplatin; pem, pemetrexed; PP, cisplatin plus pemetrexed.

**Table S3.** Any-grade adverse events occurring in ≥10% of patients and Grade 3/4 adverse events occurring in ≥2% of patients in either treatment arm

| **Patients, n (%)** | **APP  (n=82)** | | **PP  (n=81)** | |
| --- | --- | --- | --- | --- |
|  | **Any grade** | **Grade 3/4** | **Any grade** | **Grade 3/4** |
| Decreased neutrophil count | 66 (80.5) | 41 (50.0) | 45 (55.6) | 21 (25.9) |
| Decreased white blood cell count | 59 (72.0) | 18 (22.0) | 46 (56.8) | 10 (12.3) |
| Increased alanine aminotransferase | 29 (35.4) | 0 | 18 (22.2) | 2 (2.5) |
| Increased aspartate aminotransferase | 29 (35.4) | 0 | 18 (22.2) | 1 (1.2) |
| Decreased platelet count | 20 (24.4) | 6 (7.3) | 14 (17.3) | 1 (1.2) |
| Increased blood creatinine | 11 (13.4) | 0 | 12 (14.8) | 0 |
| Decreased weight | 11 (13.4) | 0 | 8 (9.9) | 1 (1.2) |
| Increased gamma-glutamyltransferase | 9 (11.0) | 1 (1.2) | 6 (7.4) | 1 (1.2) |
| Weight increased | 9 (11.0) | 0 | 3 (3.7) | 0 |
| Anemia | 62 (75.6) | 13 (15.9) | 55 (67.9) | 8 (9.9) |
| Leukopenia | 9 (11.0) | 2 (2.4) | 7 (8.6) | 0 |
| Neutropenia | 6 (7.3) | 2 (2.4) | 10 (12.3) | 3 (3.7) |
| Nausea | 40 (48.8) | 0 | 41 (50.6) | 1 (1.2) |
| Constipation | 35 (42.7) | 1 (1.2) | 24 (29.6) | 0 |
| Vomiting | 22 (26.8) | 1 (1.2) | 24 (29.6) | 2 (2.5) |
| Diarrhea | 15 (18.3) | 2 (2.4) | 8 (9.9) | 0 |
| Malaise | 21 (25.6) | 1 (1.2) | 16 (19.8) | 2 (2.5) |
| Pyrexia | 22 (26.8) | 0 | 8 (9.9) | 0 |
| Asthenia | 13 (15.9) | 0 | 7 (8.6) | 0 |
| Fatigue | 13 (15.9) | 1 (1.2) | 5 (6.2) | 3 (3.7) |
| Decreased appetite | 25 (30.5) | 4 (4.9) | 25 (30.9) | 1 (1.2) |
| Hypoalbuminemia | 9 (11) | 0 | 11 (13.6) | 1 (1.2) |
| Hyponatremia | 11 (13.4) | 7 (8.5) | 8 (9.9) | 5 (6.2) |
| Productive cough | 14 (17.1) | 0 | 7 (8.6) | 0 |
| Cough | 11 (13.4) | 0 | 7 (8.6) | 0 |
| Hiccups | 9 (11.0) | 0 | 9 (11.1) | 0 |
| Dyspnea | 11 (13.4) | 0 | 5 (6.2) | 0 |
| Upper respiratory tract infection | 16 (19.5) | 2 (2.4) | 8 (9.9) | 0 |
| Dizziness | 11 (13.4) | 0 | 5 (6.2) | 0 |
| Rash | 11 (13.4) | 2 (2.4) | 8 (9.9) | 1 (1.2) |
| Hyperthyroidism | 10 (12.2) | 0 | 1 (1.2) | 0 |
| Hypokalemia | 8 (9.8) | 4 (4.9) | 5 (6.2) | 0 |
| Hypertension | 6 (7.3) | 2 (2.4) | 3 (3.7) | 2 (2.5) |
| Pneumonia | 6 (7.3) | 1 (1.2) | 4 (4.9) | 3 (3.7) |
| Hypophosphatemia | 5 (6.1) | 2 (2.4) | 1 (1.2) | 0 |

Percentages are based on N in the column headings. Data cutoff was July 18, 2019. APP, atezolizumab plus cisplatin plus pemetrexed; PP, cisplatin plus pemetrexed.

**Table S4.** Adverse events of special interest among treated patients, by arm

| **Patients, n (%)** | **APP  (n=82)** | | **PP  (n=81)** | |
| --- | --- | --- | --- | --- |
|  | **Any grade** | **Grade 3/4** | **Any grade** | **Grade 3/4** |
| Immune-mediated hepatitis | 44 (53.7) | 3 (3.7) | 25 (30.9) | 3 (3.7) |
| Laboratory result abnormalities | 43 (52.4) | 2 (2.4) | 25 (30.9)^a^ | 3 (3.7) |
| Diagnosis | 2 (2.4) | 1 (1.2) | 1 (1.2) | 0 |
| Immune-mediated rash | 18 (22.0) | 3 (3.7) | 11 (13.6) | 1 (1.2) |
| Immune-mediated hypothyroidism | 13 (15.9) | 0 | 1 (1.2) | 0 |
| Immune-mediated hyperthyroidism | 10 (12.2) | 0 | 1 (1.2) | 0 |
| Immune-mediated pneumonitis | 4 (4.9) | 1 (1.2) | 2 (2.5) | 1 (1.2) |
| Infusion-related reactions | 3 (3.7) | 1 (1.2) | 0 | 0 |
| Immune-mediated adrenal insufficiency | 1 (1.2) | 0 | 0 | 0 |
| Immune-mediated autoimmune hemolytic anemia | 1 (1.2) | 0 | 0 | 0 |
| Immune-mediated meningitis | 1 (1.2) | 0 | 0 | 0 |
| Immune-mediated meningoencephalitis | 1 (1.2) | 0 | 0 | 0 |
| Immune-mediated ocular inflammatory toxic | 0 | 0 | 1 (1.2) | 0 |

Terms were specified by the sponsor, regardless of investigator-assessed causality. No patients reported cases of pancreatitis, severe cutaneous reactions, nephritis, colitis, diabetes mellitus, vasculitis, myocarditis, systemic immune activation, encephalitis, hypophysitis, myositis and rhabdomyolysis. Data cutoff was July 18, 2019. AE, adverse event; APP, atezolizumab plus cisplatin plus pemetrexed; PP, cisplatin plus pemetrexed.

^a^ One Grade 5 event was observed.

**Figure S1.** Investigator-assessed progression-free survival (PFS) in key patient subgroups. Data cutoff was July 18, 2019. APP, atezolizumab plus cisplatin plus pemetrexed; ECOG PS, Eastern Cooperative Oncology Group performance status; PP, cisplatin plus pemetrexed.

**
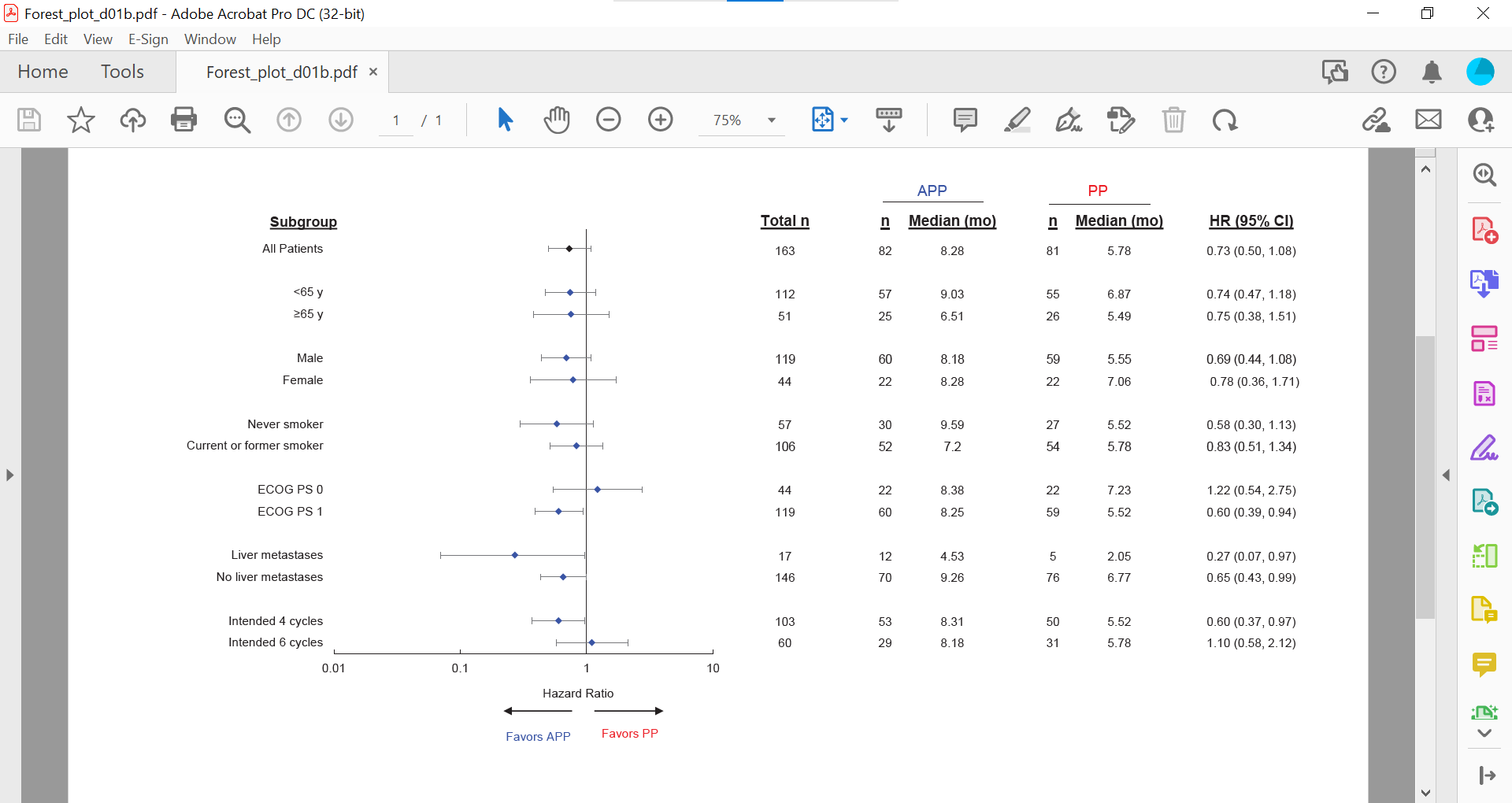
**
